# Supplementary material for: Determinants of carbon release from the active layer and permafrost deposits on the Tibetan Plateau
Source: Nat Commun. 2016 Oct 5;7:13046. doi: 10.1038/ncomms13046 (PMC5059472; doi:10.1038/ncomms13046)
Supplement: Supplementary Information — Supplementary Figures 1-5, Supplementary Tables 1-10 and Supplementary Reference [file ncomms13046-s1.pdf]

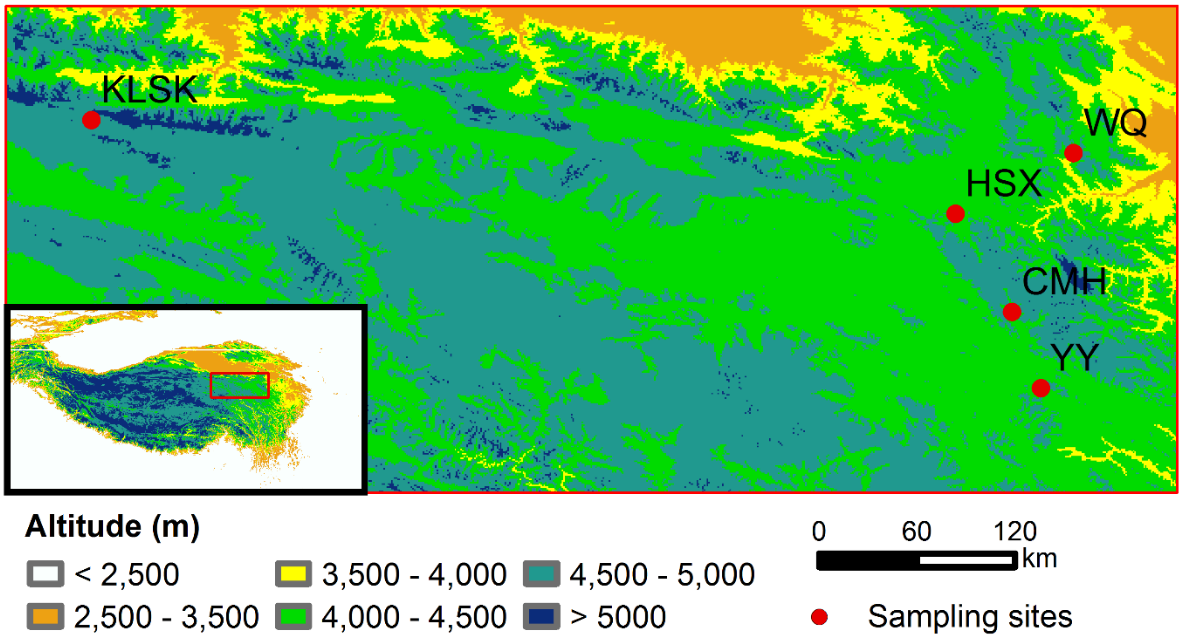

Supplementary Figure 1. A map of permafrost sampling locations with site names shown next to the closed red circles. YY: Youyun, Maqin County; CMH: Changmahe, Maqin County; HSX: Huashixia, Maduo County; WQ: Wenquan, Xinghai County; KLSK: Kunlunshankou, Geermu.

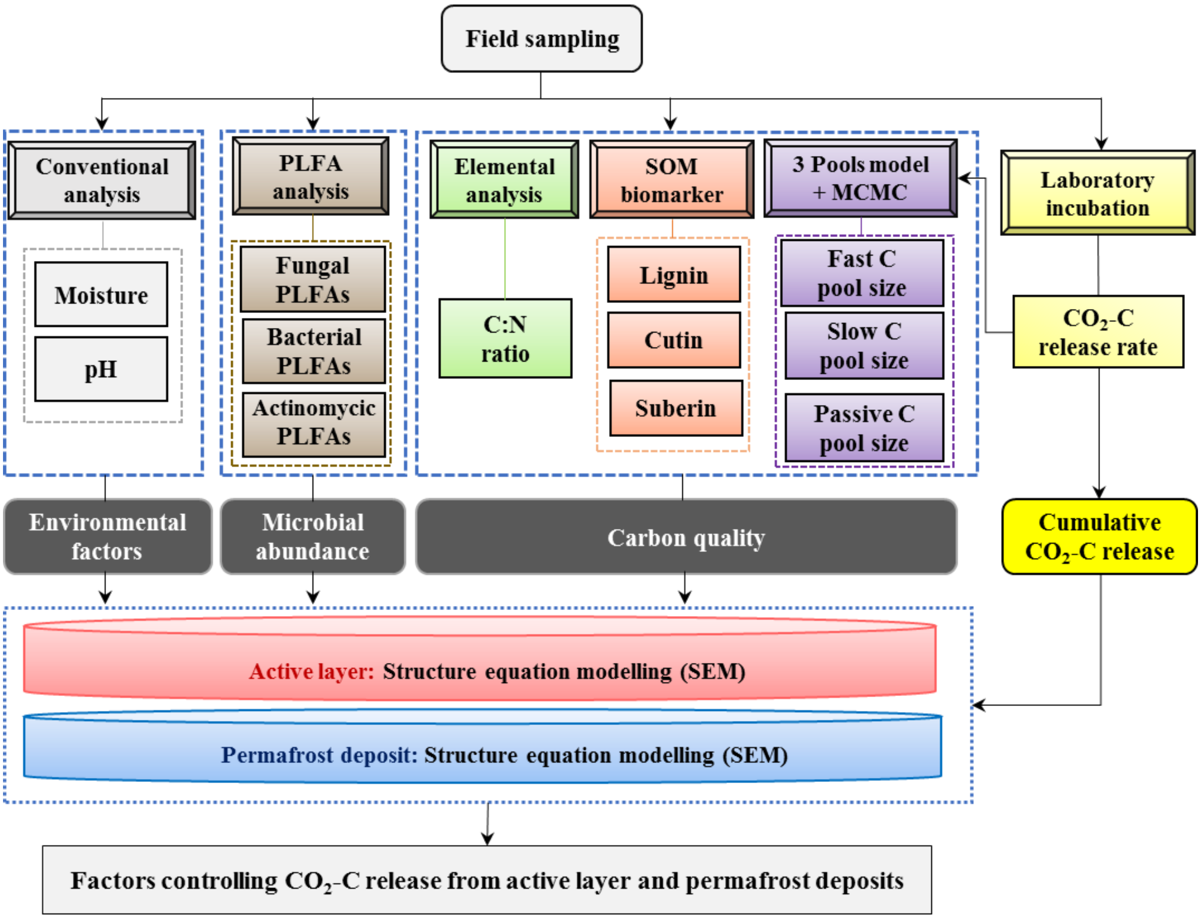

Supplementary Figure 2. Methodology used to determine the factor controlling CO_2-C release from the active layer and permafrost deposits. Three types of proxies (soil C:N ratio, the relative abundance of SOM components derived from biomarker analysis, and the pool sizes for C fractions derived from a three-pool model) were used to quantify C quality. After quantifying the SOC quality, we combined these variables with environmental factors (*i.e.*, soil moisture, and pH) and microbial abundance variables (*i.e.*, fungal PLFAs, actinomycete PLFAs, and microbial PLFAs) together to explore the relative importance of these factors in regulating the CO_2-C release on the basis of structure equation modelling (SEM).

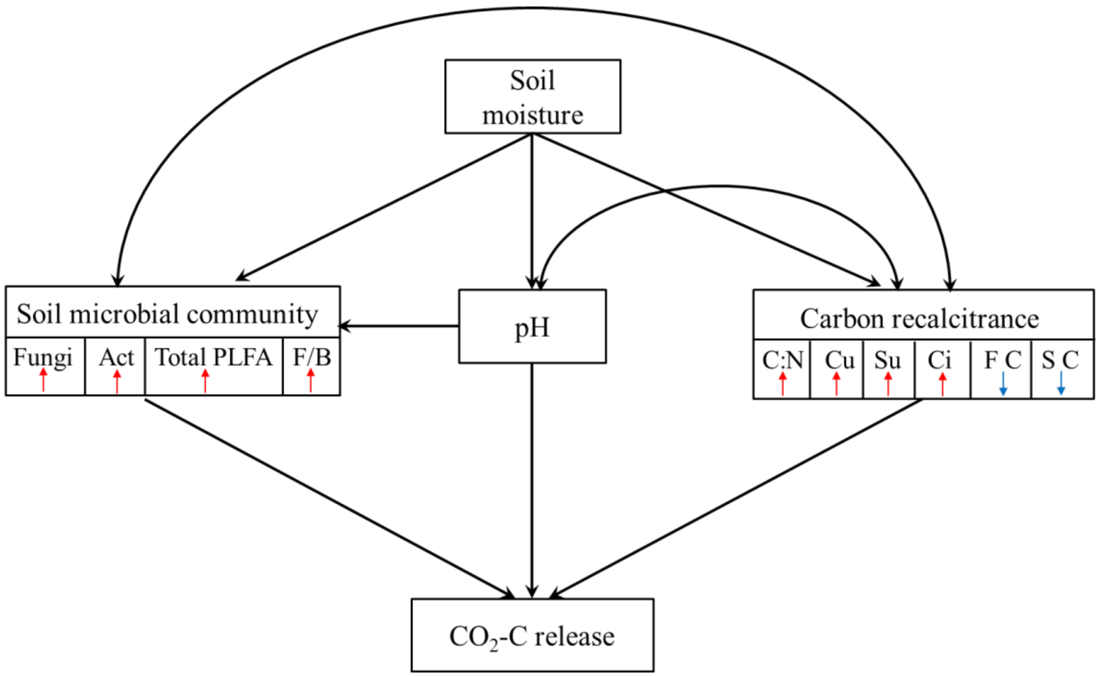

Supplementary Figure 3. A priori model of the effects of soil moisture, pH, carbon recalcitrance and soil microbial community on the CO_2-C release. Double-layer rectangles represent the first component from the PCA conducted with soil microbial community and carbon recalcitrance. Soil microbial community includes total PLFAs, fungal PLFAs (Fungi), actinomycete PLFAs (Act) and the fungi/bacteria ratio (F/B) as indicated by PLFA analysis; Carbon recalcitrance includes C:N, the cutin-derived components (Cu), suberin-derived components (Su), lignin cinnamyl units (Ci), fast C pool size (FC) and slow C pool size (SC). Double-headed arrows represent the covariance between related variables. Arrow direction indicates the hypothesized direction of causation. The red symbol "↑" and blue symbol "↓" indicate a positive or negative relationship between the variables and the first component from the PCA, respectively.

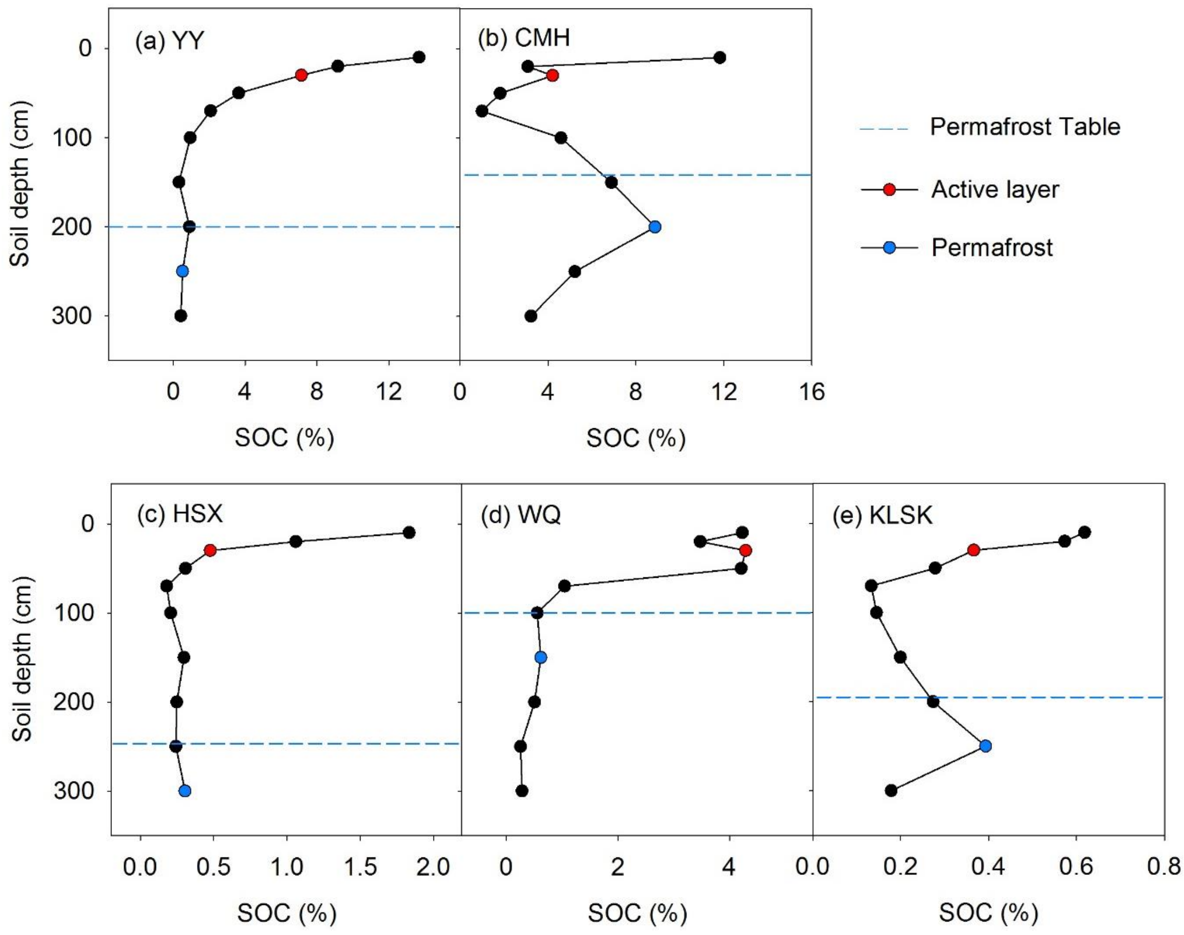

Supplementary Figure 4. Vertical distributions of soil organic carbon concentrations at the five study sites. The short blue dashed line indicates the position of the permafrost table in the borehole. Red and blue dots indicate the samples from the active layer and permafrost deposits that were used for laboratory incubation, respectively.

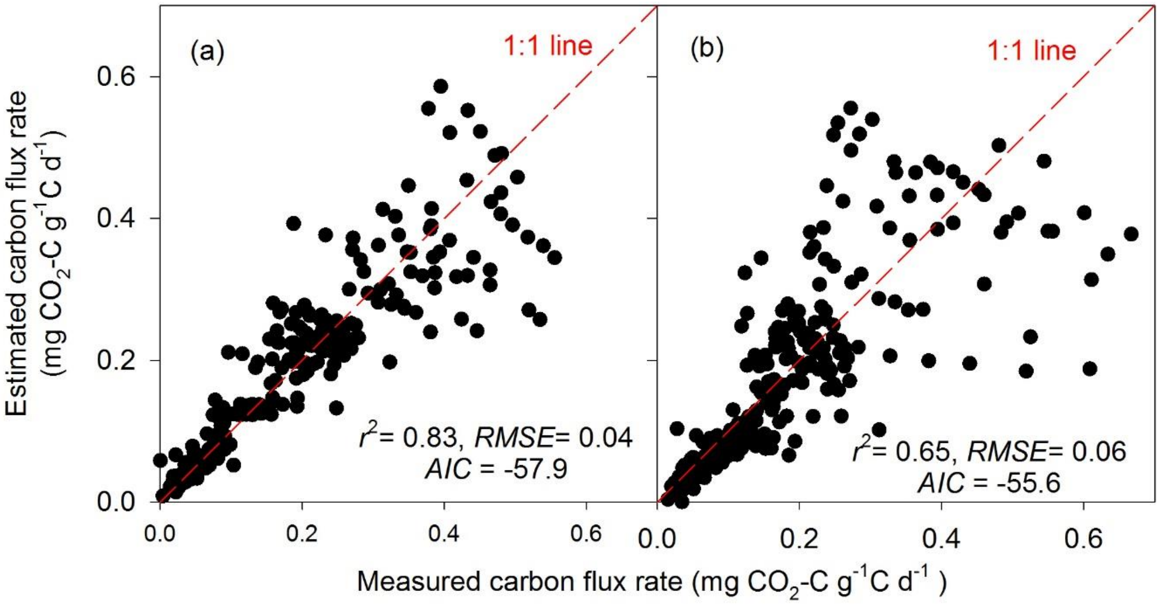

Supplementary Figure 5. Comparison of three-pool (a) and two-pool (b) model performance using data from our 80-day incubation study.

Supplementary Table 1. Characteristics of the sampling sites used in this study.

Site name	Coordinates	Altitude (m)	MAT (°C)	MAP (mm)	EVI	Active layer thickness (cm)*	Ecosystem type	Dominant species
YY	99.302°E, 34.144°N	4344	-2.6	480	0.23	80-175	Swamp meadow	*Kobresia tibetica*
CMH	99.143°E, 34.566°N	4417	-2.9	419	0.15	80-110	Swamp meadow	*K. tibetica*
HSX	98.832°E, 35.107°N	4283	-1.8	343	0.16	210-250	Alpine meadow	*K. kansuensis, K. tibetica*
WQ	99.482°E, 35.441°N	4092	0.17	347	0.17	85-90	Alpine meadow	*K. pygmaca*
KLSK	94.066°E, 35.625°N	4763	-0.15	183	0.05	150-180	Alpine steppe	*Littledalea racemosa*

MAT, mean annual air temperature; MAP, mean annual precipitation; EVI, enhanced vegetation index. YY: Youyun, Maqin county; CMH: Changmahe, Maqin County; HSX: Huashixia, Maduo County; WQ: Wenquan, Xinghai County; KLSK: Kunlunshankou, Geermu.

*Measured in August 2013.

Supplementary Table 2. Environmental characteristics of the active layer and permafrost samples within the soil profile at five sites.

Site name	Layer	Sample size	Sample depth (cm)	Soil moisture (%)	SOC (g kg^{-1})	Bulk density (g cm^{-3})	C:N	NH_4^+-N (mg N kg^{-1})	NO_3^--N (mg N kg^{-1})	pH	Clay+Silt (%)
YY	AL	3	20-30	65.2 [55.0-80.0]	70.9 [54.0-86.0]	1.0 [1.0-1.0]	12.9 [12.5-13.2]	32.1 [25.2-37.5]	4.6 [3.3-5.7]	6.8 [6.7-6.9]	33.2 [32.3-34.5]
	PF	3	200-250	17.6 [14.7-20.4]	5.4 [5.0-6.2]	1.4 [1.3-1.4]	7.9 [7.6-8.6]	12.1 [11.5-12.6]	3.6 [3.2-3.9]	8.3 [8.3-8.4]	27.3 [25.3-28.4]
CMH	AL	3	20-30	49.9 [47.8-52.1]	61.3 [36.5-75.4]	1.0 [1.0-1.0]	12.1 [11.6-12.6]	8.1 [7.9-8.2]	2.3 [1.8-2.6]	7.6 [7.4-7.9]	29.8 [25.5-32.4]
	PF	3	150-200	77.9 [74.4-81.0]	79.9 [62.8-91.2]	1.0 [1.0-1.0]	14.1 [13.9-14.2]	68.8 [57.0-75.5]	3.9 [2.7-5.2]	7.5 [7.4-7.6]	38.4 [37.8-38.8]
HSX	AL	3	20-30	13.0 [11.3-14.3]	6.0 [4.3-7.6]	1.3 [1.3-1.4]	6.2 [5.4-7.0]	3.5 [3.2-3.7]	3.3 [2.5-4.1]	8.3 [8.2-8.4]	33.4 [29.5-37.3]
	PF	3	250-300	13.2 [11.7-14.3]	2.2 [2.1-2.3]	1.5 [1.5-1.5]	5.8 [5.6-6.0]	3.2 [3.2-3.3]	1.9 [1.2-2.3]	8.6 [8.5-8.6]	37.4 [35.7-38.3]
WQ	AL	3	20-30	34.2 [32.5-36.1]	44.4 [41.0-48.0]	1.0 [1.0-1.0]	11.4 [11.2-11.7]	3.7 [3.5-3.8]	1.6 [1.4-1.6]	7.2 [7.1-7.3]	47.0 [46.4-47.4]
	PF	3	100-150	25.9 [21.1-28.6]	6.0 [4.8-6.7]	1.3 [1.3-1.4]	7.0 [6.4-7.5]	3.4 [3.2-3.7]	1.4 [0.8-1.7]	8.0 [8.0-8.1]	35.3 [32.8-37.7]
KLSK	AL	3	20-30	4.70 [3.92-5.12]	3.9 [3.8-4.1]	1.4 [1.4-1.4]	10.5 [9.8-11.0]	3.0 [2.7-3.1]	1.7 [1.4-1.9]	8.6 [8.6-8.7]	19.9 [16.2-22.4]
	PF	3	200-250	20.5 [19.7-21.1]	6.1 [5.0-7.0]	1.3 [1.3-1.4]	17.6 [16.2-20.0]	7.5 [7.1-8.2]	1.8 [1.5-2.0]	8.0 [8.0-8.1]	40.3 [37.7-42.6]

YY: Youyun, Maqin County; CMH: Changmahe, Maqin County; HSX: Huashixia, Maduo County; WQ: Wenquan, Xinghai County; KLSK: Kunlunshankou, Geermu. Layers: AL, active layer; PF, permafrost deposits. The interquartile range is presented in square brackets.

Supplementary Table 3. Chemical characteristics of dissolved organic matter in the active layer and permafrost deposits across five study sites.

Site name	Sample size	Layer	DOC (mg C kg^{-1} soil)	DOC (mg C kg^{-1} OC)	TDN (mg C kg^{-1} soil)	$SUVA_{254}$ (kg mg^{-1} OC m^{-1})
YY	3	AL	300.9 [289.7-321.5]	4.7 [3.9-5.6]	33.7 [25.2-37.5]	1.0 [0.9-1.1]
	3	PF	58.1 [45.5-75.3]	10.1 [8.4-12.1]	10.5 [8.6-13.0]	0.7 [0.7-0.7]
CMH	3	AL	125.3 [119.3-133.8]	2.6 [2.2-3.4]	21.5 [18.9-23.9]	0.9 [0.8-1.0]
	3	PF	334.6 [253.1-380.9]	4.1 [4.0-4.3]	56.4 [51.4-59.5]	0.4 [0.4-0.4]
HSX	3	AL	46.5 [34.2-59.8]	7.8 [7.6-7.9]	9.8 [7.0-12.3]	1.2 [1.0-1.3]
	3	PF	12.4 [12.3-12.7]	5.6 [5.4-5.8]	4.9 [4.5-5.0]	0.8 [0.6-0.9]
WQ	3	AL	200.8 [180.7-237.3]	4.7 [3.9-5.8]	21.8 [20.3-24.2]	1.0 [0.9-1.1]
	3	PF	34.1 [30.2-40.5]	5.8 [4.7-6.5]	6.1 [5.5-6.6]	0.9 [0.8-0.9]
KLSK	3	AL	19.0 [17.9-20.4]	4.8 [4.7-5.0]	4.3 [4.2-4.4]	1.7 [1.6-1.9]
	3	PF	19.6 [16.9-22.5]	2.1 [2.0-2.3]	8.0 [7.2-8.9]	0.9 [0.8-1.0]

YY: Youyun, Maqin County; CMH: Changmahe, Maqin County; HSX: Huashixia, Maduo County; WQ: Wenquan, Xinghai County; KLSK: Kunlunshankou, Geermu. AL, active layer; PF, permafrost deposits. DOC, dissolved organic carbon; TDN, total dissolved nitrogen; $SUVA_{254}$, specific ultraviolet absorbance at 254 nm. The interquartile range is presented in square brackets.

Supplementary Table 4. ANOVA table for cumulative CO_2 production from different sites, soil layers and temperature treatments.

	NumDF	DenDF	*F*	*P*
(Intercept)	1	20	473.2852	<0.0001
Site	4	10	40.9115	<0.0001
Layer	1	10	88.7433	<0.0001
Temp	1	20	10.188	0.0046
Site × Layer	4	10	18.5230	0.0001
Site × Temp	4	20	4.634	0.0082
Layer × Temp	1	20	1.656	0.2129
Layer × Temp × Site	4	20	0.647	0.6356

NumDF: numerator degrees of freedom; DenDF: denominator degrees of freedom.

Supplementary Table 5. The mean residence time of different C pools for all of the samples.

Site	Layer	Mean residence time (years)		
		Fast C	Slow C	Passive C
YY	AL	0.09 [0.06, 0.10]	19.3 [13.6, 22.6]	>950
	PF	0.22 [0.19, 0.26]	5.65 [5.63, 5.66]	>800
CMH	AL	0.61 [0.17, 0.85]	20.6 [16.6, 24.1]	>1900
	PF	0.42 [0.17, 0.58]	36.6 [30.7, 44.7]	>2700
HSX	AL	0.56 [0.25, 0.80]	5.80 [5.62, 5.91]	>1000
	PF	0.10 [0.09, 0.10]	5.72 [5.67, 5.79]	>950
WQ	AL	0.13 [0.11, 0.14]	14.7 [14.0, 15.8]	>1100
	PF	0.50 [0.27, 0.62]	5.86 [5.71, 5.97]	>1100
KLSK	AL	0.54 [0.26,0.69]	5.85 [5.80, 5.92]	>2700
	PF	0.22 [0.17, 0.30]	7.68 [6.07, 8.73]	>950

YY: Youyun, Maqin County; CMH: Changmahe, Maqin County; HSX: Huashixia, Maduo County; WQ: Wenquan, Xinghai County; KLSK: Kunlunshankou, Geermu. AL, active layer; PF, permafrost deposits. The interquartile range is presented in square brackets. The interquartile range is presented in square brackets.

Supplementary Table 6. Contribution of different C pools to cumulative CO_2-C release for 80-day and 10200-day incubation.

Site	Layer	Contribution to total C loss from the 80-day incubation			Contribution to total C loss from the 10200-day projection ~ 85 years in situ[a]		
		Fast C %	Slow C %	Passive C %	Fast C %	Slow C %	Passive C %
YY	AL	18.3 [4.22, 25.4]	66.6 [56.5, 80.8]	15.1 [12.4, 18.1]	0.48 [0.10, 0.67]	56.6 [49.7, 60.3]	42.9 [39.5, 49.6]
	PF	32.5 [31.4, 34.1]	64.2 [62.2, 65.4]	3.21 [2.49, 3.68]	3.45 [3.32, 3.61]	70.3 [67.3, 73.4]	26.3 [23.0, 29.4]
CMH	AL	26.7 [16.3, 39.2]	63.5 [57.6, 70.6]	9.81 [3.19, 13.1]	0.74 [0.57, 0.93]	80.9 [76.5, 89.5]	18.3 [9.60, 23.0]
	PF	61.5 [55.6, 65.7]	34.6 [30.6, 40.0]	3.92 [3.64, 4.34]	2.36 [2.18, 2.71]	82.3 [81.6, 83.2]	15.4 [14.6, 15.8]
HSX	AL	22.1 [14.1, 30.8]	74.8 [67.2, 81.5]	3.14 [1.99, 4.40]	2.99 [2.29, 3.87]	76.6 [71.2, 79.3]	20.4 [16.8, 26.5]
	PF	28.2 [27.6, 29.4]	70.6 [69.8, 71.0]	1.21 [0.82, 1.56]	3.96 [3.74, 4.21]	77.8 [73.5, 82.5]	18.2 [13.3, 22.8]
WQ	AL	11.8 [10.3, 13.9]	70.9 [69.6, 71.7]	17.3 [14.5, 20.0]	0.49 [0.26, 0.64]	58.0 [52.7, 62.3]	41.5 [37.4, 46.7]
	PF	28.6 [19.6, 38.7]	68.7 [59.3, 77.6]	2.70 [2.04, 3.25]	3.27 [2.92, 3.91]	75.0 [71.8, 77.3]	21.7 [18.7, 25.3]
KLSK	AL	31.1 [23.0, 43.8]	67.0 [54.7, 75.2]	1.94 [1.46, 2.63]	3.51 [2.77, 4.39]	80.7 [76.2, 84.0]	15.8 [11.6, 21.1]
	PF	29.1 [22.6, 33.9]	67.8 [64.9, 73.3]	3.06 [1.21, 4.13]	2.78 [2.13, 3.95]	77.9 [76.8, 78.5]	19.3 [19.3, 19.4]

[a] We assumed that soils would be thawed for only 4 months per year and stay at a constant temperature of 5 °C. Therefore, 1 year of incubation represents in situ conditions for 3 years. Thus, the next 85 years until the year 2100 represents incubation for approximately 28.3 years (~10,200 days). YY: Youyun, Maqin County; CMH: Changmahe, Maqin County; HSX: Huashixia, Maduo County; WQ: Wenquan, Xinghai County; KLSK: Kunlunshankou, Geermu. AL, active layer; PF, permafrost deposits. The interquartile range is presented in square brackets.

Supplementary Table 7. Prior parameter ranges for C pool partitioning coefficients (f_i) and decay rates (k_i).

Parameter	Description	lower limit	upper limit
f_1	fraction of fast C pool	0	0.01
f_2	fraction of slow C pool	0	0.20
k_1	decay rate of fast C pool	0	0.05
k_2	decay rate of slow C pool	0	0.0009[a]
k_3	decay rate of passive C pool	0	9×10^{-5}[a]

C pool fractions are unit less, decay rates are day^{-1}.

[a] in soils at Changmahe, Maqin County (CMH) the upper limit of k_2 was set to 1×10^{-4}, and the upper limit of k_3 was set to 5×10^{-5}.

Supplementary Table 8. Maximum likelihood estimates (MLEs) of posterior probability density functions of model parameters for all soil samples.

Site	Sample	Parameter				
		k_1 $(x10^{-2})$	k_2 $(x10^{-5})$	k_3 $(x10^{-6})$	f_1 $(x10^{-3})$	f_2 $(x10^{-2})$
YY	1-1	1.97 [1.84, 2.11]	4.15 [3.97, 4.61]	2.28 [1.82, 2.68]	1.61 [1.56, 1.67]	12.4 [9.55, 14.8]
YY	1-2	1.41 [1.32, 1.50]	48.7 [47.9, 49.6]	47.3 [45.8, 49.6]	9.57 [9.35, 9.82]	19.5 [19.4, 19.8]
YY	1-3	3.39 [2.00, 4.76]	11.2 [6.01, 14.4]	23.2 [20.3, 26.8]	0.40 [0.19, 0.36]	6.56 [4.37, 8.45]
YY	1-4	0.75 [0.66, 0.84]	48.6 [48.5, 49.4]	47.1 [45.3, 49.2]	9.22 [8.78, 9.83]	19.6 [19.5, 19.8]
YY	1-5	1.59 [0.12, 3.44]	13.3 [10.6, 17.1]	11.0 [6.76, 14.9]	2.86 [0.21, 5.93]	7.96 [6.74, 10.8]
YY	1-6	0.60 [0.52, 0.67]	48.3 [47.8, 49.4]	46.3 [44.0, 48.6]	8.86 [8.43, 9.38]	19.2 [18.7, 19.6]
CMH	2-1	0.03 [0.01, 0.05]	4.91 [1.53, 8.82]	26.4 [24.1, 30.3]	1.74 [0.53, 2.00]	12.5 [8.44, 16.7]
CMH	2-2	0.30 [0.27, 0.32]	8.26 [4.13, 9.78]	10.0 [5.38, 14.3]	3.03 [2.64, 3.47]	11.7 [7.68, 15.7]
CMH	2-3	1.84 [1.49, 2.11]	2.18 [0.58, 2.40]	31.1 [30.3, 31.5]	1.10 [1.01, 1.20]	7.38 [5.62, 9.94]
CMH	2-4	1.22 [1.16, 1.29]	0.92 [0.89, 0.96]	4.73 [4.60, 4.91]	2.18 [2.13, 2.27]	16.6 [15.0, 18.3]
CMH	2-5	1.29 [1.19, 1.37]	0.53 [0.44, 0.62]	1.99 [1.65, 2.33]	4.19 [3.91, 4.41]	5.74 [2.36, 8.44]
CMH	2-6	2.45 [2.41, 2.55]	0.19 [0.13, 0.22]	4.39 [4.05, 4.76]	2.38 [2.34, 2.41]	7.11 [5.66, 9.57]
HSX	3-1	2.73 [2.53, 2.85]	45.6 [44.9, 49.3]	44.8 [44.2, 47.3]	9.54 [9.23, 9.88]	19.0 [18.8, 19.7]
HSX	3-2	2.80 [2.45, 3.14]	45.7 [45.4, 49.3]	46.9 [45.2, 49.1]	9.49 [9.28, 9.82]	18.7 [17.9, 19.7]
HSX	3-3	0.29 [0.09, 0.41]	80.3 [66.0, 89.1]	39.0 [34.7, 45.0]	2.39 [0.56, 3.99]	15.9 [13.9, 18.1]
HSX	3-4	2.19 [2.06, 2.32]	47.8 [47.2, 49.3]	45.8 [43.6, 48.1]	9.41 [9.12, 9.87]	18.4 [17.7, 19.5]
HSX	3-5	0.22 [0.05, 0.23]	35.0 [30.1, 39.2]	34.2 [27.6, 41.8]	4.28 [0.94, 7.56]	15.6 [13.6, 17.8]
HSX	3-6	2.53 [2.47, 2.59]	48.2 [47.3, 49.7]	46.5 [45.5, 48.3]	9.61 [9.49, 9.89]	19.2 [18.6, 19.9]
WQ	4-1	1.71 [1.49, 1.92]	6.11 [4.07, 8.45]	12.0 [8.78, 14.2]	0.56 [0.45, 0.68]	14.2 [12.9, 15.8]
WQ	4-2	1.05 [0.96, 1.13]	37.0 [32.7, 42.4]	30.3 [22.2, 40.2]	9.17 [8.78, 9.66]	15.0 [13.0, 17.4]
WQ	4-3	2.00 [1.35, 2.57]	16.5 [12.4, 19.8]	4.11 [1.67, 4.95]	0.12 [0.07, 0.14]	8.04 [6.61, 9.24]
WQ	4-4	0.24 [0.10, 0.33]	40.8 [37.6, 44.5]	38.9 [34.1, 44.6]	3.54 [1.20, 5.58]	16.4 [15.1, 18.0]
WQ	4-5	0.85 [0.39, 1.11]	6.91 [4.20, 9.29]	10.2 [6.55, 13.3]	0.41 [0.16, 0.68]	12.0 [10.5, 14.2]
WQ	4-6	0.76 [0.68, 0.84]	45.1 [42.9, 47.9]	44.6 [41.7, 47.9]	8.75 [8.13, 9.35]	18.0 [17.3, 19.3]
KLSK	5-1	0.27 [0.11, 0.34]	40.8 [37.4, 45.1]	33.9 [27.0, 42.5]	3.91 [1.09, 6.92]	15.3 [13.3, 17.4]

KLSK	5-2	4.21 [4.07, 4.40]	3.14 [1.72, 4.95]	28.8 [25.3, 31.6]	0.67 [0.61, 0.73]	11.8 [9.98, 14.1]
KLSK	5-3	1.19 [1.10, 1.27]	45.6 [44.7, 48.2]	39.2 [34.5, 45.5]	7.96 [7.18, 8.44]	16.7 [15.4, 17.8]
KLSK	5-4	0.53 [0.50, 0.56]	47.6 [46.3, 49.4]	45.6 [43.5, 48.3]	9.34 [8.93, 9.77]	19.2 [18.9, 19.8]
KLSK	5-5	1.60 [1.46, 1.62]	40.3 [36.7, 47.8]	38.1 [32.2, 46.3]	7.46 [6.84, 7.88]	18.1 [17.0, 19.1]
KLSK	5-6	0.04 [0.03, 0.04]	37.3 [32.2, 43.3]	85.5 [77.5, 92.3]	2.43 [1.34, 3.37]	14.4 [12.1, 16.7]

YY: Youyun, Maqin County; CMH: Changmahe, Maqin County; HSX: Huashixia, Maduo County; WQ: Wenquan, Xinghai County; KLSK: Kunlunshankou, Geermu. AL: active layer; PF: permafrost deposits. f_1 and f_2 represent the fractions of the fast and slow pools, respectively, and k_1, k_2, and k_3 represent the decay rates of fast, slow and passive pools, respectively. The interquartile range is presented in square brackets.

[a] We assumed that soils would be thawed for only 4 months per year and stay at a constant temperature of 5 °C. Therefore, 1 year of incubation represents in situ conditions for 3 years. Thus, the next 85 years until the year 2100 represents incubation for approximately 28.3 years (~10200 days).

Supplementary Table 9. Comparison of estimated parameters and CO_2-C release using two datasets with different incubation durations. The data were extracted from [1].

Site	Layer [a]	Depth (m)	Parameters and carbon loss [b]	Based on the 85 days' incubation	Based on the 390 days' incubation
Zelenyi Mys	AL	0.1	k_1	3.79 [3.20, 4.52]	3.84 [3.57, 4.64]
			k_2 ($\times 10^{-3}$)	1.76 [0.55, 2.35]	1.87 [0.60, 2.73]
			k_3 ($\times 10^{-5}$)	2.31 [1.68, 2.95]	2.33 [1.17, 3.35]
			f_1 ($\times 10^{-2}$)	1.15 [0.58, 1.67]	1.16 [0.59, 1.59]
			f_2 ($\times 10^{-2}$)	9.45 [3.05, 12.9]	11.5 [3.47, 13.1]
			Carbon loss for 85-day (mg C g^{-1} SOC)	19.3 [13.4, 24.2]	20.4 [14.6, 25.2]
			Carbon loss for 10200-day (mg C g^{-1} SOC)	282.8 [215.8, 350.6]	288.0 [193.7, 382.4]
	PF	2.0	k_1	4.10 [3.74, 4.65]	4.24 [3.93, 4.69]
			k_2 ($\times 10^{-3}$)	5.56 [3.97, 7.11]	6.69 [5.27, 8.26]
			k_3 ($\times 10^{-5}$)	1.68 [0.74, 2.46]	1.24 [0.05, 1.82]
			f_1 ($\times 10^{-2}$)	1.76 [0.94, 2.46]	1.90 [1.06, 2.61]
			f_2 ($\times 10^{-2}$)	3.25 [2.29, 3.49]	2.85 [2.15, 2.85]
			Carbon loss for 85-day (mg C g^{-1} SOC)	28.8 [20.6, 35.9]	30.1 [21.8, 37.1]
			Carbon loss for 10200-day (mg C g^{-1} SOC)	194.8 [122.6, 260.1]	156.6 [94.0, 206.9]

[a] AL: active layer; PF: permafrost deposit. The interquartile range is presented in square brackets.

[b] f_1 and f_2 are the fractions of fast and slow pools, k_1, k_2, k_3 are the decay rates of fast, slow and passive pools, respectively. We assumed that soils would be thawed for only 4 months per year and stay at a constant temperature of 5 °C. Then 1 year of incubation represents in situ conditions for 3 years. Thus, the next 85 years till 2100 represents incubation for roughly 28.3 years (≈10200 days).

Supplementary Table 10. Results of the principal components analysis (PCA) of soil C recalcitrance and soil microorganisms from the active layer (a) and permafrost deposits (b).

(a) Active layer

**Variable**	**PC1**
**Carbon recalcitrance**	
C:N	0.979***
Cutin-derived compounds (mg g^{-1} OC)	0.982***
Suberin-derived compounds (mg g^{-1} OC)	0.901***
Cinnamyl (mg g^{-1} OC)	0.788***
Fast C pool size (% of initial SOC)	-0.787***
Slow C pool size (% of initial SOC)	-0.782***
**Cumulative (%)**	**76.44**
**Soil microorganisms**	
Total PLFA (mg g^{-1} OC)	0.948***
Fungal PLFAs (mg g^{-1} OC)	0.991***
Actinomycic PLFAs (mg g^{-1} OC)	0.970***
F/ B	0.838***
**Cumulative (%)**	**88.09**

(b) Permafrost deposits

**Variable**	**PC1**
**Carbon recalcitrance**	
C:N	0.51**
Cutin-derived compounds (mg g^{-1} OC)	0.92***
Suberin-derived compounds (mg g^{-1} OC)	0.87***
Lignin cinnamyl unit (mg g^{-1} OC)	0.93***
Fast C pool size (% of initial SOC)	-0.74***
Slow C pool size (% of initial SOC)	-0.79***
**Cumulative (%)**	**68.07**
**Soil microorganisms**	
Total PLFAs (mg g^{-1}OC)	0.992***
Fungal PLFAs (mg g^{-1}OC)	0.988***
Actinomycic PLFAs (mg g^{-1}OC)	0.940***
**Cumulative (%)**	**94.08**

Supplementary References

1 Dutta, K., Schuur, E. A. G., Neff, J. C. & Zimov, S. A. Potential carbon release from permafrost soils of Northeastern Siberia. *Global Change Biol.* **12**, 2336-2351 (2006).
